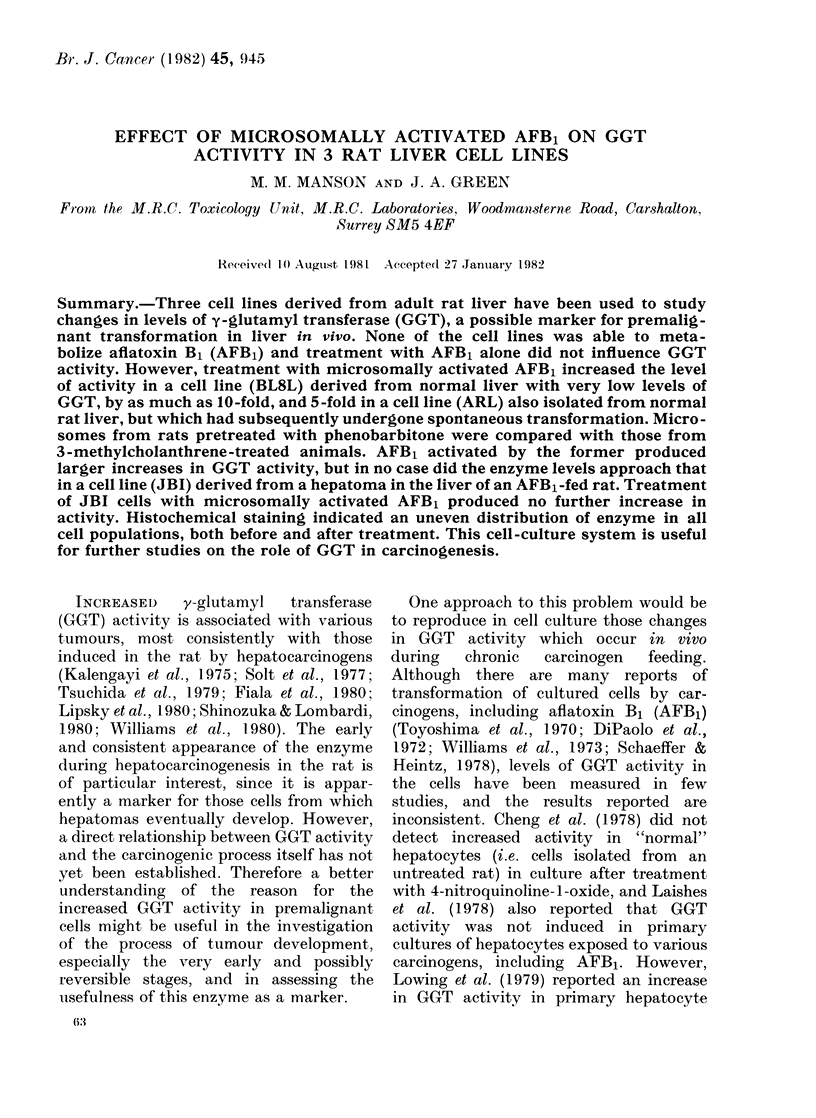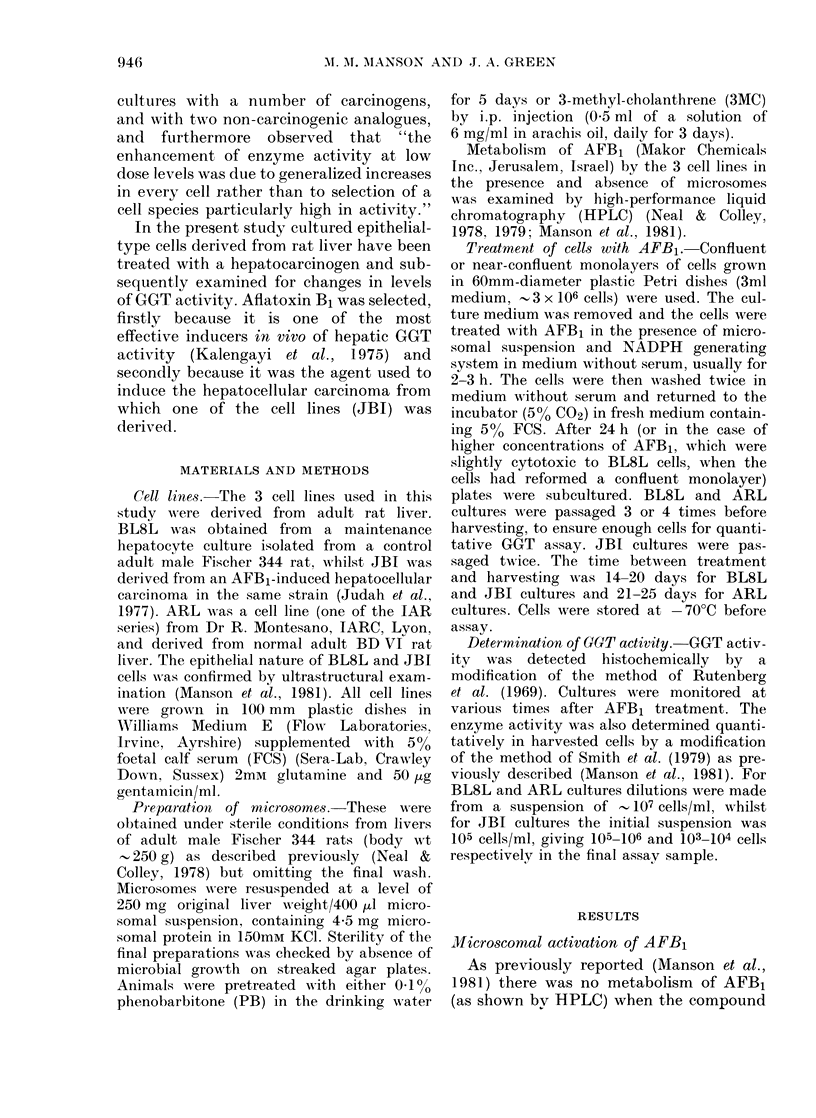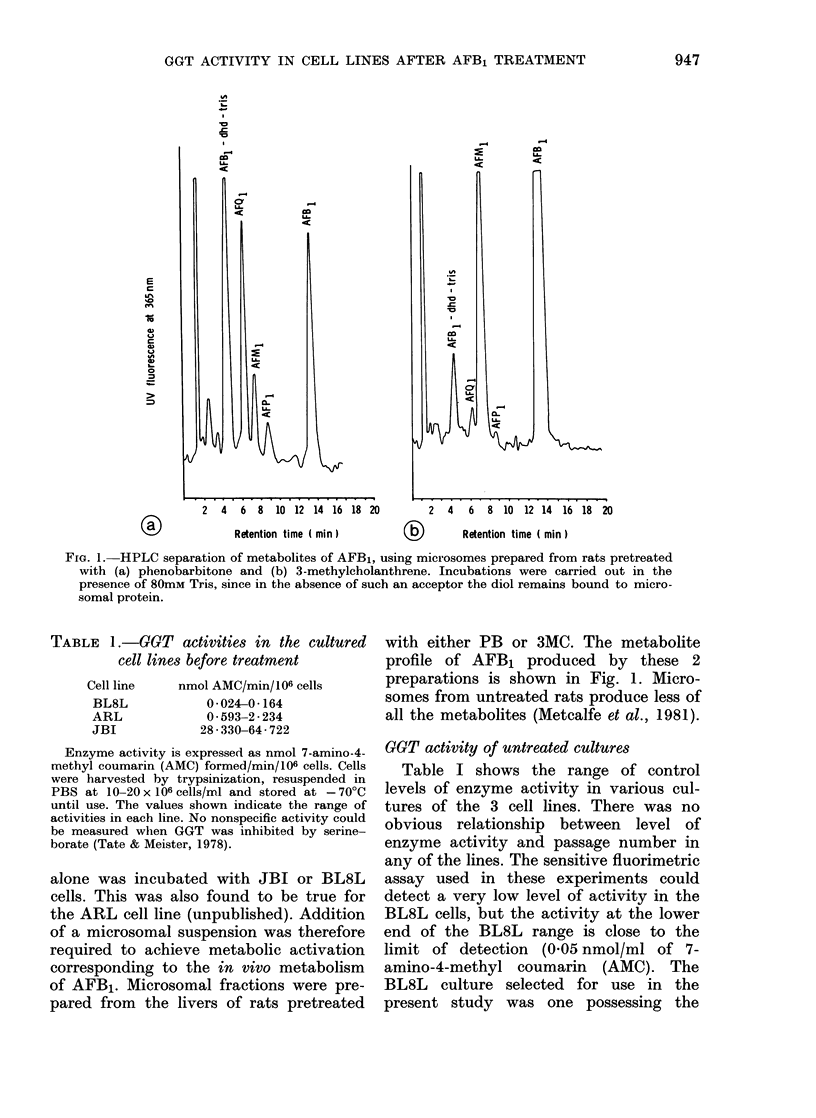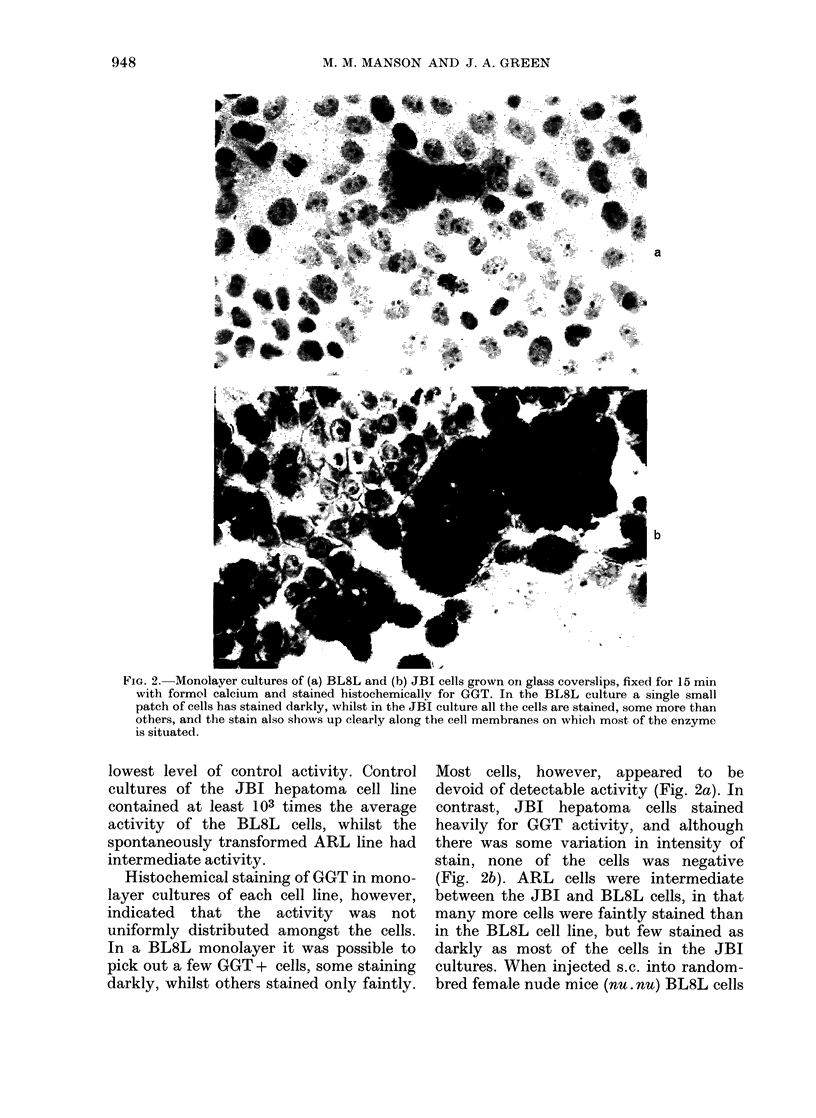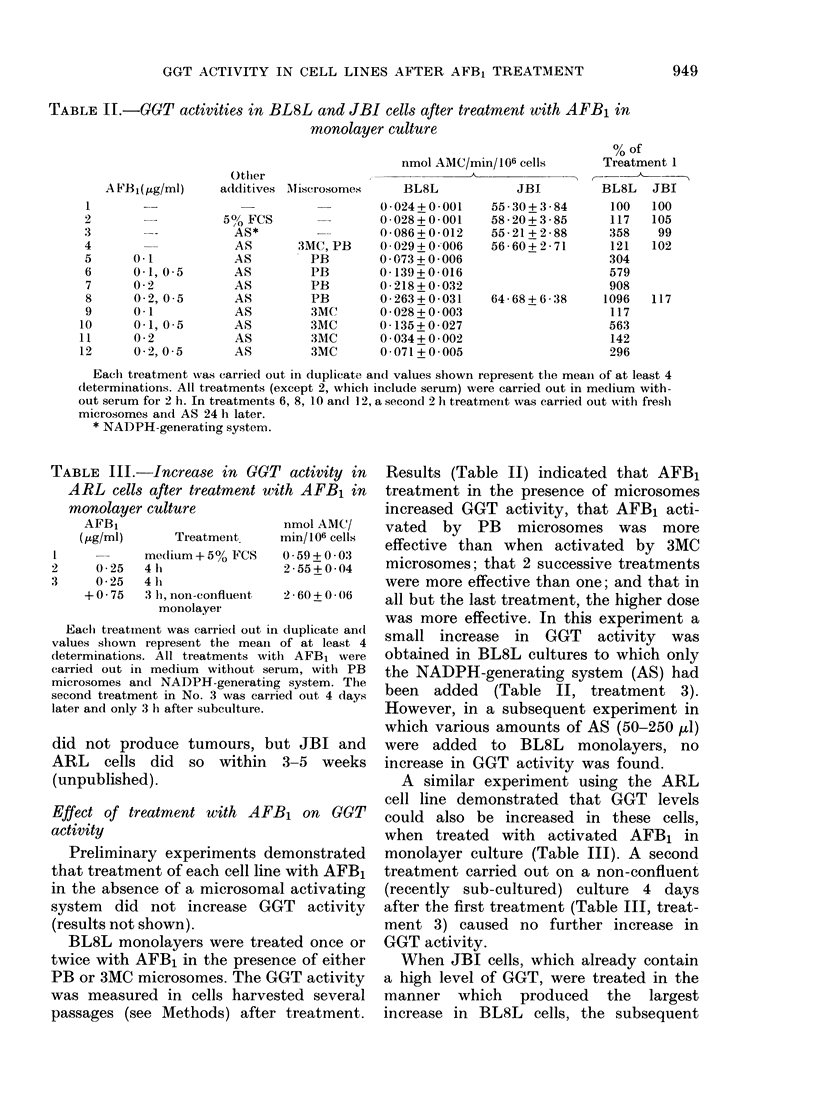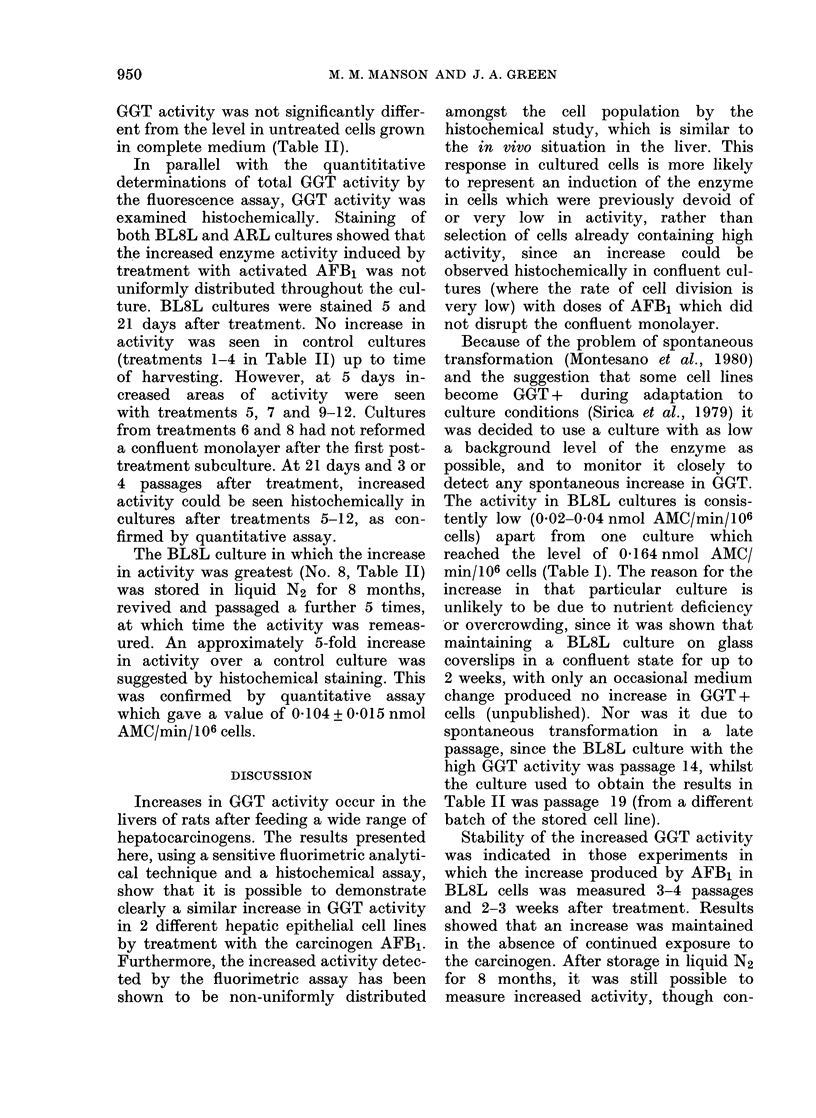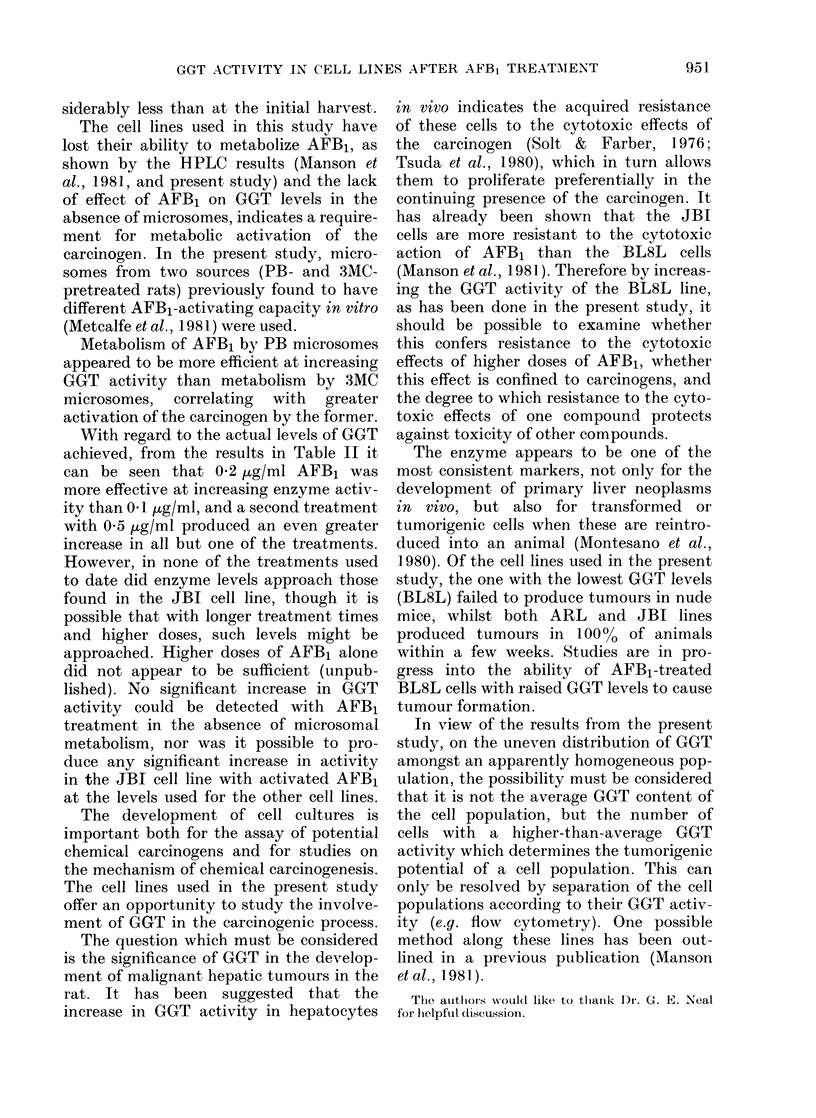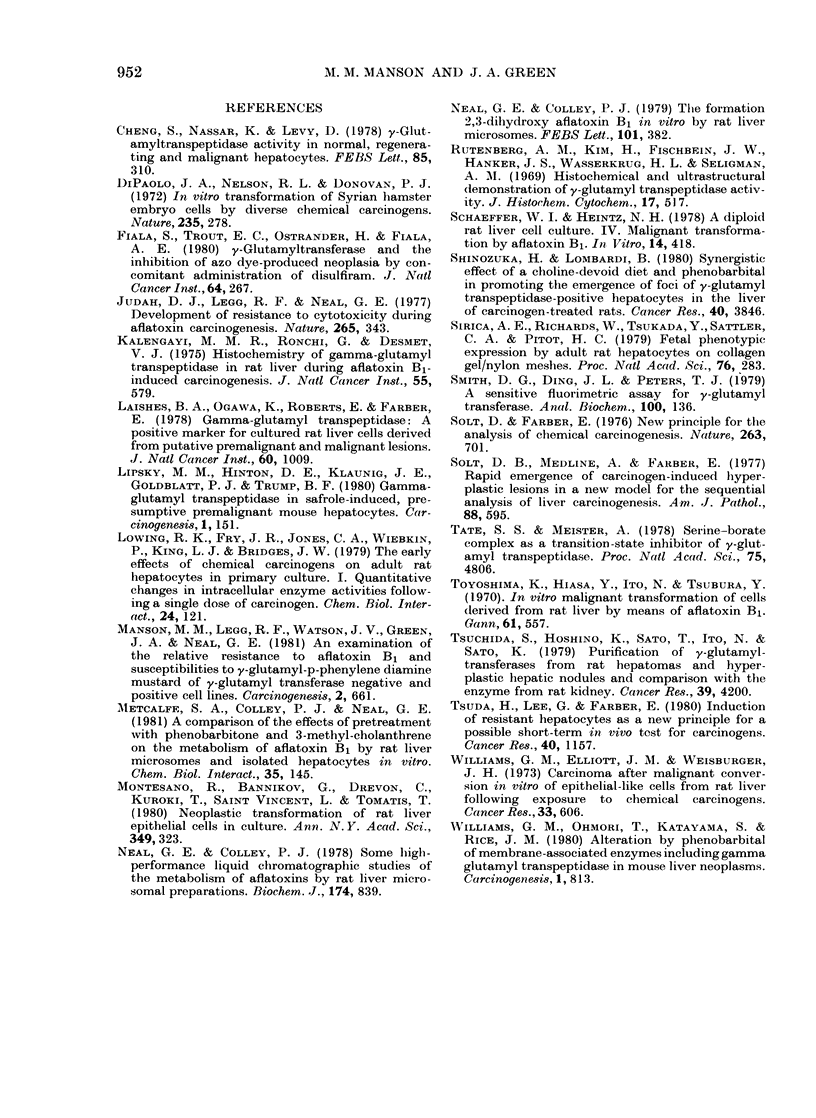# Effect of microsomally activated AFB1 on GGT activity in 3 rat liver cell lines.

**DOI:** 10.1038/bjc.1982.147

**Published:** 1982-06

**Authors:** M. M. Manson, J. A. Green

## Abstract

**Images:**


*Br. J. Cancer* (1982) **45**, 945

### EFFECT OF MICROSOMALLY ACTIVATED AFB$_1$ ON GGT ACTIVITY IN 3 RAT LIVER CELL LINES

M. M. MANSON AND J. A. GREEN

*From the M.R.C. Toxicology Unit, M.R.C. Laboratories, Woodmansterne Road, Carshalton, Surrey SM5 4EF*

Received 10 August 1981 Accepted 27 January 1982

**Summary.—Three cell lines derived from adult rat liver have been used to study changes in levels of γ-glutamyl transferase (GGT), a possible marker for premalignant transformation in liver *in vivo*. None of the cell lines was able to metabolize aflatoxin B$_1$ (AFB$_1$) and treatment with AFB$_1$ alone did not influence GGT activity. However, treatment with microsomally activated AFB$_1$ increased the level of activity in a cell line (BL8L) derived from normal liver with very low levels of GGT, by as much as 10-fold, and 5-fold in a cell line (ARL) also isolated from normal rat liver, but which had subsequently undergone spontaneous transformation. Microsomes from rats pretreated with phenobarbitone were compared with those from 3-methylcholanthrene-treated animals. AFB$_1$ activated by the former produced larger increases in GGT activity, but in no case did the enzyme levels approach that in a cell line (JBI) derived from a hepatoma in the liver of an AFB$_1$-fed rat. Treatment of JBI cells with microsomally activated AFB$_1$ produced no further increase in activity. Histochemical staining indicated an uneven distribution of enzyme in all cell populations, both before and after treatment. This cell-culture system is useful for further studies on the role of GGT in carcinogenesis.**

INCREASED γ-glutamyl transferase (GGT) activity is associated with various tumours, most consistently with those induced in the rat by hepatocarcinogens (Kalengayi *et al.*, 1975; Solt *et al.*, 1977; Tsuchida *et al.*, 1979; Fiala *et al.*, 1980; Lipsky *et al.*, 1980; Shinozuka & Lombardi, 1980; Williams *et al.*, 1980). The early and consistent appearance of the enzyme during hepatocarcinogenesis in the rat is of particular interest, since it is apparently a marker for those cells from which hepatomas eventually develop. However, a direct relationship between GGT activity and the carcinogenic process itself has not yet been established. Therefore a better understanding of the reason for the increased GGT activity in premalignant cells might be useful in the investigation of the process of tumour development, especially the very early and possibly reversible stages, and in assessing the usefulness of this enzyme as a marker.

One approach to this problem would be to reproduce in cell culture those changes in GGT activity which occur *in vivo* during chronic carcinogen feeding. Although there are many reports of transformation of cultured cells by carcinogens, including aflatoxin B$_1$ (AFB$_1$) (Toyoshima *et al.*, 1970; DiPaolo *et al.*, 1972; Williams *et al.*, 1973; Schaeffer & Heintz, 1978), levels of GGT activity in the cells have been measured in few studies, and the results reported are inconsistent. Cheng *et al.* (1978) did not detect increased activity in "normal" hepatocytes (*i.e.* cells isolated from an untreated rat) in culture after treatment with 4-nitroquinoline-1-oxide, and Laishes *et al.* (1978) also reported that GGT activity was not induced in primary cultures of hepatocytes exposed to various carcinogens, including AFB$_1$. However, Lowing *et al.* (1979) reported an increase in GGT activity in primary hepatocyte cultures with a number of carcinogens, and with two non-carcinogenic analogues, and furthermore observed that "the enhancement of enzyme activity at low dose levels was due to generalized increases in every cell rather than to selection of a cell species particularly high in activity."

In the present study cultured epithelial-type cells derived from rat liver have been treated with a hepatocarcinogen and subsequently examined for changes in levels of GGT activity. Aflatoxin $B_1$ was selected, firstly because it is one of the most effective inducers *in vivo* of hepatic GGT activity (Kalengayi *et al.*, 1975) and secondly because it was the agent used to induce the hepatocellular carcinoma from which one of the cell lines (JBI) was derived.

### MATERIALS AND METHODS

*Cell lines*.—The 3 cell lines used in this study were derived from adult rat liver. BL8L was obtained from a maintenance hepatocyte culture isolated from a control adult male Fischer 344 rat, whilst JBI was derived from an $AFB_1$-induced hepatocellular carcinoma in the same strain (Judah *et al.*, 1977). ARL was a cell line (one of the IAR series) from Dr R. Montesano, IARC, Lyon, and derived from normal adult BD VI rat liver. The epithelial nature of BL8L and JBI cells was confirmed by ultrastructural examination (Manson *et al.*, 1981). All cell lines were grown in 100 mm plastic dishes in Williams Medium E (Flow Laboratories, Irvine, Ayrshire) supplemented with 5% foetal calf serum (FCS) (Sera-Lab, Crawley Down, Sussex) 2mM glutamine and 50 $\mu$g gentamicin/ml.

*Preparation of microsomes*.—These were obtained under sterile conditions from livers of adult male Fischer 344 rats (body wt ~250 g) as described previously (Neal & Colley, 1978) but omitting the final wash. Microsomes were resuspended at a level of 250 mg original liver weight/400 $\mu$l microsomal suspension, containing 4·5 mg microsomal protein in 150mM KCl. Sterility of the final preparations was checked by absence of microbial growth on streaked agar plates. Animals were pretreated with either 0·1% phenobarbitone (PB) in the drinking water for 5 days or 3-methyl-cholanthrene (3MC) by i.p. injection (0·5 ml of a solution of 6 mg/ml in arachis oil, daily for 3 days).

Metabolism of $AFB_1$ (Makor Chemicals Inc., Jerusalem, Israel) by the 3 cell lines in the presence and absence of microsomes was examined by high-performance liquid chromatography (HPLC) (Neal & Colley, 1978, 1979; Manson *et al.*, 1981).

*Treatment of cells with $AFB_1$*.—Confluent or near-confluent monolayers of cells grown in 60mm-diameter plastic Petri dishes (3ml medium, $\sim 3 \times 10^6$ cells) were used. The culture medium was removed and the cells were treated with $AFB_1$ in the presence of microsomal suspension and NADPH generating system in medium without serum, usually for 2–3 h. The cells were then washed twice in medium without serum and returned to the incubator (5% $CO_2$) in fresh medium containing 5% FCS. After 24 h (or in the case of higher concentrations of $AFB_1$, which were slightly cytotoxic to BL8L cells, when the cells had reformed a confluent monolayer) plates were subcultured. BL8L and ARL cultures were passaged 3 or 4 times before harvesting, to ensure enough cells for quantitative GGT assay. JBI cultures were passaged twice. The time between treatment and harvesting was 14–20 days for BL8L and JBI cultures and 21–25 days for ARL cultures. Cells were stored at −70°C before assay.

*Determination of GGT activity*.—GGT activity was detected histochemically by a modification of the method of Rutenberg *et al.* (1969). Cultures were monitored at various times after $AFB_1$ treatment. The enzyme activity was also determined quantitatively in harvested cells by a modification of the method of Smith *et al.* (1979) as previously described (Manson *et al.*, 1981). For BL8L and ARL cultures dilutions were made from a suspension of $\sim 10^7$ cells/ml, whilst for JBI cultures the initial suspension was $10^5$ cells/ml, giving $10^5$–$10^6$ and $10^3$–$10^4$ cells respectively in the final assay sample.

### RESULTS

### *Microscomal activation of $AFB_1$*

As previously reported (Manson *et al.*, 1981) there was no metabolism of $AFB_1$ (as shown by HPLC) when the compound

FIG. 1.—HPLC separation of metabolites of $AFB_1$, using microsomes prepared from rats pretreated with (a) phenobarbitone and (b) 3-methylcholanthrene. Incubations were carried out in the presence of 80mM Tris, since in the absence of such an acceptor the diol remains bound to microsomal protein.

TABLE 1.—*GGT activities in the cultured cell lines before treatment*

| Cell line | nmol AMC/min/$10^6$ cells |
|---|---|
| BL8L | 0·024–0·164 |
| ARL | 0·593–2·234 |
| JBI | 28·330–64·722 |

Enzyme activity is expressed as nmol 7-amino-4-methyl coumarin (AMC) formed/min/$10^6$ cells. Cells were harvested by trypsinization, resuspended in PBS at 10–20 × $10^6$ cells/ml and stored at −70°C until use. The values shown indicate the range of activities in each line. No nonspecific activity could be measured when GGT was inhibited by serine–borate (Tate & Meister, 1978).

alone was incubated with JBI or BL8L cells. This was also found to be true for the ARL cell line (unpublished). Addition of a microsomal suspension was therefore required to achieve metabolic activation corresponding to the *in vivo* metabolism of $AFB_1$. Microsomal fractions were prepared from the livers of rats pretreated with either PB or 3MC. The metabolite profile of $AFB_1$ produced by these 2 preparations is shown in Fig. 1. Microsomes from untreated rats produce less of all the metabolites (Metcalfe *et al.*, 1981).

### *GGT activity of untreated cultures*

Table I shows the range of control levels of enzyme activity in various cultures of the 3 cell lines. There was no obvious relationship between level of enzyme activity and passage number in any of the lines. The sensitive fluorimetric assay used in these experiments could detect a very low level of activity in the BL8L cells, but the activity at the lower end of the BL8L range is close to the limit of detection (0·05 nmol/ml of 7-amino-4-methyl coumarin (AMC). The BL8L culture selected for use in the present study was one possessing the

FIG. 2.—Monolayer cultures of (a) BL8L and (b) JBI cells grown on glass coverslips, fixed for 15 min with formol calcium and stained histochemically for GGT. In the BL8L culture a single small patch of cells has stained darkly, whilst in the JBI culture all the cells are stained, some more than others, and the stain also shows up clearly along the cell membranes on which most of the enzyme is situated.

lowest level of control activity. Control cultures of the JBI hepatoma cell line contained at least $10^3$ times the average activity of the BL8L cells, whilst the spontaneously transformed ARL line had intermediate activity.

Histochemical staining of GGT in monolayer cultures of each cell line, however, indicated that the activity was not uniformly distributed amongst the cells. In a BL8L monolayer it was possible to pick out a few GGT+ cells, some staining darkly, whilst others stained only faintly. Most cells, however, appeared to be devoid of detectable activity (Fig. 2*a*). In contrast, JBI hepatoma cells stained heavily for GGT activity, and although there was some variation in intensity of stain, none of the cells was negative (Fig. 2*b*). ARL cells were intermediate between the JBI and BL8L cells, in that many more cells were faintly stained than in the BL8L cell line, but few stained as darkly as most of the cells in the JBI cultures. When injected s.c. into randombred female nude mice (*nu.nu*) BL8L cells

TABLE II.—*GGT activities in BL8L and JBI cells after treatment with $AFB_1$ in monolayer culture*

| | $AFB_1$ (μg/ml) | Other additives | Miscrosomes | nmol AMC/min/$10^6$ cells BL8L | JBI | % of Treatment 1 BL8L | JBI |
|---|---|---|---|---|---|---|---|
| 1 | — | — | — | 0·024±0·001 | 55·30±3·84 | 100 | 100 |
| 2 | — | 5% FCS | — | 0·028±0·001 | 58·20±3·85 | 117 | 105 |
| 3 | — | AS* | — | 0·086±0·012 | 55·21±2·88 | 358 | 99 |
| 4 | — | AS | 3MC, PB | 0·029±0·006 | 56·60±2·71 | 121 | 102 |
| 5 | 0·1 | AS | PB | 0·073±0·006 | | 304 | |
| 6 | 0·1, 0·5 | AS | PB | 0·139±0·016 | | 579 | |
| 7 | 0·2 | AS | PB | 0·218±0·032 | | 908 | |
| 8 | 0·2, 0·5 | AS | PB | 0·263±0·031 | 64·68±6·38 | 1096 | 117 |
| 9 | 0·1 | AS | 3MC | 0·028±0·003 | | 117 | |
| 10 | 0·1, 0·5 | AS | 3MC | 0·135±0·027 | | 563 | |
| 11 | 0·2 | AS | 3MC | 0·034±0·002 | | 142 | |
| 12 | 0·2, 0·5 | AS | 3MC | 0·071±0·005 | | 296 | |

Each treatment was carried out in duplicate and values shown represent the mean of at least 4 determinations. All treatments (except 2, which include serum) were carried out in medium without serum for 2 h. In treatments 6, 8, 10 and 12, a second 2 h treatment was carried out with fresh microsomes and AS 24 h later.

* NADPH-generating system.

TABLE III.—*Increase in GGT activity in ARL cells after treatment with $AFB_1$ in monolayer culture*

| | $AFB_1$ (μg/ml) | Treatment | nmol AMC/min/$10^6$ cells |
|---|---|---|---|
| 1 | — | medium + 5% FCS | 0·59±0·03 |
| 2 | 0·25 | 4 h | 2·55±0·04 |
| 3 | 0·25 | 4 h | |
| | +0·75 | 3 h, non-confluent monolayer | 2·60±0·06 |

Each treatment was carried out in duplicate and values shown represent the mean of at least 4 determinations. All treatments with $AFB_1$ were carried out in medium without serum, with PB microsomes and NADPH-generating system. The second treatment in No. 3 was carried out 4 days later and only 3 h after subculture.

did not produce tumours, but JBI and ARL cells did so within 3–5 weeks (unpublished).

### *Effect of treatment with $AFB_1$ on GGT activity*

Preliminary experiments demonstrated that treatment of each cell line with $AFB_1$ in the absence of a microsomal activating system did not increase GGT activity (results not shown).

BL8L monolayers were treated once or twice with $AFB_1$ in the presence of either PB or 3MC microsomes. The GGT activity was measured in cells harvested several passages (see Methods) after treatment. Results (Table II) indicated that $AFB_1$ treatment in the presence of microsomes increased GGT activity, that $AFB_1$ activated by PB microsomes was more effective than when activated by 3MC microsomes; that 2 successive treatments were more effective than one; and that in all but the last treatment, the higher dose was more effective. In this experiment a small increase in GGT activity was obtained in BL8L cultures to which only the NADPH-generating system (AS) had been added (Table II, treatment 3). However, in a subsequent experiment in which various amounts of AS (50–250 μl) were added to BL8L monolayers, no increase in GGT activity was found.

A similar experiment using the ARL cell line demonstrated that GGT levels could also be increased in these cells, when treated with activated $AFB_1$ in monolayer culture (Table III). A second treatment carried out on a non-confluent (recently sub-cultured) culture 4 days after the first treatment (Table III, treatment 3) caused no further increase in GGT activity.

When JBI cells, which already contain a high level of GGT, were treated in the manner which produced the largest increase in BL8L cells, the subsequent

GGT activity was not significantly different from the level in untreated cells grown in complete medium (Table II).

In parallel with the quantititative determinations of total GGT activity by the fluorescence assay, GGT activity was examined histochemically. Staining of both BL8L and ARL cultures showed that the increased enzyme activity induced by treatment with activated $AFB_1$ was not uniformly distributed throughout the culture. BL8L cultures were stained 5 and 21 days after treatment. No increase in activity was seen in control cultures (treatments 1–4 in Table II) up to time of harvesting. However, at 5 days increased areas of activity were seen with treatments 5, 7 and 9–12. Cultures from treatments 6 and 8 had not reformed a confluent monolayer after the first post-treatment subculture. At 21 days and 3 or 4 passages after treatment, increased activity could be seen histochemically in cultures after treatments 5–12, as confirmed by quantitative assay.

The BL8L culture in which the increase in activity was greatest (No. 8, Table II) was stored in liquid $N_2$ for 8 months, revived and passaged a further 5 times, at which time the activity was remeasured. An approximately 5-fold increase in activity over a control culture was suggested by histochemical staining. This was confirmed by quantitative assay which gave a value of $0{\cdot}104 \pm 0{\cdot}015$ nmol AMC/min/$10^6$ cells.

### DISCUSSION

Increases in GGT activity occur in the livers of rats after feeding a wide range of hepatocarcinogens. The results presented here, using a sensitive fluorimetric analytical technique and a histochemical assay, show that it is possible to demonstrate clearly a similar increase in GGT activity in 2 different hepatic epithelial cell lines by treatment with the carcinogen $AFB_1$. Furthermore, the increased activity detected by the fluorimetric assay has been shown to be non-uniformly distributed amongst the cell population by the histochemical study, which is similar to the *in vivo* situation in the liver. This response in cultured cells is more likely to represent an induction of the enzyme in cells which were previously devoid of or very low in activity, rather than selection of cells already containing high activity, since an increase could be observed histochemically in confluent cultures (where the rate of cell division is very low) with doses of $AFB_1$ which did not disrupt the confluent monolayer.

Because of the problem of spontaneous transformation (Montesano *et al.*, 1980) and the suggestion that some cell lines become GGT+ during adaptation to culture conditions (Sirica *et al.*, 1979) it was decided to use a culture with as low a background level of the enzyme as possible, and to monitor it closely to detect any spontaneous increase in GGT. The activity in BL8L cultures is consistently low ($0{\cdot}02$–$0{\cdot}04$ nmol AMC/min/$10^6$ cells) apart from one culture which reached the level of $0{\cdot}164$ nmol AMC/min/$10^6$ cells (Table I). The reason for the increase in that particular culture is unlikely to be due to nutrient deficiency or overcrowding, since it was shown that maintaining a BL8L culture on glass coverslips in a confluent state for up to 2 weeks, with only an occasional medium change produced no increase in GGT+ cells (unpublished). Nor was it due to spontaneous transformation in a late passage, since the BL8L culture with the high GGT activity was passage 14, whilst the culture used to obtain the results in Table II was passage 19 (from a different batch of the stored cell line).

Stability of the increased GGT activity was indicated in those experiments in which the increase produced by $AFB_1$ in BL8L cells was measured 3–4 passages and 2–3 weeks after treatment. Results showed that an increase was maintained in the absence of continued exposure to the carcinogen. After storage in liquid $N_2$ for 8 months, it was still possible to measure increased activity, though considerably less than at the initial harvest.

The cell lines used in this study have lost their ability to metabolize $AFB_1$, as shown by the HPLC results (Manson *et al.*, 1981, and present study) and the lack of effect of $AFB_1$ on GGT levels in the absence of microsomes, indicates a requirement for metabolic activation of the carcinogen. In the present study, microsomes from two sources (PB- and 3MC-pretreated rats) previously found to have different $AFB_1$-activating capacity *in vitro* (Metcalfe *et al.*, 1981) were used.

Metabolism of $AFB_1$ by PB microsomes appeared to be more efficient at increasing GGT activity than metabolism by 3MC microsomes, correlating with greater activation of the carcinogen by the former.

With regard to the actual levels of GGT achieved, from the results in Table II it can be seen that 0·2 μg/ml $AFB_1$ was more effective at increasing enzyme activity than 0·1 μg/ml, and a second treatment with 0·5 μg/ml produced an even greater increase in all but one of the treatments. However, in none of the treatments used to date did enzyme levels approach those found in the JBI cell line, though it is possible that with longer treatment times and higher doses, such levels might be approached. Higher doses of $AFB_1$ alone did not appear to be sufficient (unpublished). No significant increase in GGT activity could be detected with $AFB_1$ treatment in the absence of microsomal metabolism, nor was it possible to produce any significant increase in activity in the JBI cell line with activated $AFB_1$ at the levels used for the other cell lines.

The development of cell cultures is important both for the assay of potential chemical carcinogens and for studies on the mechanism of chemical carcinogenesis. The cell lines used in the present study offer an opportunity to study the involvement of GGT in the carcinogenic process.

The question which must be considered is the significance of GGT in the development of malignant hepatic tumours in the rat. It has been suggested that the increase in GGT activity in hepatocytes *in vivo* indicates the acquired resistance of these cells to the cytotoxic effects of the carcinogen (Solt & Farber, 1976; Tsuda *et al.*, 1980), which in turn allows them to proliferate preferentially in the continuing presence of the carcinogen. It has already been shown that the JBI cells are more resistant to the cytotoxic action of $AFB_1$ than the BL8L cells (Manson *et al.*, 1981). Therefore by increasing the GGT activity of the BL8L line, as has been done in the present study, it should be possible to examine whether this confers resistance to the cytotoxic effects of higher doses of $AFB_1$, whether this effect is confined to carcinogens, and the degree to which resistance to the cytotoxic effects of one compound protects against toxicity of other compounds.

The enzyme appears to be one of the most consistent markers, not only for the development of primary liver neoplasms *in vivo*, but also for transformed or tumorigenic cells when these are reintroduced into an animal (Montesano *et al.*, 1980). Of the cell lines used in the present study, the one with the lowest GGT levels (BL8L) failed to produce tumours in nude mice, whilst both ARL and JBI lines produced tumours in 100% of animals within a few weeks. Studies are in progress into the ability of $AFB_1$-treated BL8L cells with raised GGT levels to cause tumour formation.

In view of the results from the present study, on the uneven distribution of GGT amongst an apparently homogeneous population, the possibility must be considered that it is not the average GGT content of the cell population, but the number of cells with a higher-than-average GGT activity which determines the tumorigenic potential of a cell population. This can only be resolved by separation of the cell populations according to their GGT activity (*e.g.* flow cytometry). One possible method along these lines has been outlined in a previous publication (Manson *et al.*, 1981).

The authors would like to thank Dr. G. E. Neal for helpful discussion.

### REFERENCES

Cheng, S., Nassar, K. & Levy, D. (1978) γ-Glutamyltranspeptidase activity in normal, regenerating and malignant hepatocytes. *FEBS Lett.*, **85**, 310.

DiPaolo, J. A., Nelson, R. L. & Donovan, P. J. (1972) *In vitro* transformation of Syrian hamster embryo cells by diverse chemical carcinogens. *Nature*, **235**, 278.

Fiala, S., Trout, E. C., Ostrander, H. & Fiala, A. E. (1980) γ-Glutamyltransferase and the inhibition of azo dye-produced neoplasia by concomitant administration of disulfiram. *J. Natl Cancer Inst.*, **64**, 267.

Judah, D. J., Legg, R. F. & Neal, G. E. (1977) Development of resistance to cytotoxicity during aflatoxin carcinogenesis. *Nature*, **265**, 343.

Kalengayi, M. M. R., Ronchi, G. & Desmet, V. J. (1975) Histochemistry of gamma-glutamyl transpeptidase in rat liver during aflatoxin $B_1$-induced carcinogenesis. *J. Natl Cancer Inst.*, **55**, 579.

Laishes, B. A., Ogawa, K., Roberts, E. & Farber, E. (1978) Gamma-glutamyl transpeptidase: A positive marker for cultured rat liver cells derived from putative premalignant and malignant lesions. *J. Natl Cancer Inst.*, **60**, 1009.

Lipsky, M. M., Hinton, D. E., Klaunig, J. E., Goldblatt, P. J. & Trump, B. F. (1980) Gamma-glutamyl transpeptidase in safrole-induced, presumptive premalignant mouse hepatocytes. *Carcinogenesis*, **1**, 151.

Lowing, R. K., Fry, J. R., Jones, C. A., Wiebkin, P., King, L. J. & Bridges, J. W. (1979) The early effects of chemical carcinogens on adult rat hepatocytes in primary culture. I. Quantitative changes in intracellular enzyme activities following a single dose of carcinogen. *Chem. Biol. Interact.*, **24**, 121.

Manson, M. M., Legg, R. F., Watson, J. V., Green, J. A. & Neal, G. E. (1981) An examination of the relative resistance to aflatoxin $B_1$ and susceptibilities to γ-glutamyl-p-phenylene diamine mustard of γ-glutamyl transferase negative and positive cell lines. *Carcinogenesis*, **2**, 661.

Metcalfe, S. A., Colley, P. J. & Neal, G. E. (1981) A comparison of the effects of pretreatment with phenobarbitone and 3-methyl-cholanthrene on the metabolism of aflatoxin $B_1$ by rat liver microsomes and isolated hepatocytes *in vitro*. *Chem. Biol. Interact.*, **35**, 145.

Montesano, R., Bannikov, G., Drevon, C., Kuroki, T., Saint Vincent, L. & Tomatis, T. (1980) Neoplastic transformation of rat liver epithelial cells in culture. *Ann. N.Y. Acad. Sci.*, **349**, 323.

Neal, G. E. & Colley, P. J. (1978) Some high-performance liquid chromatographic studies of the metabolism of aflatoxins by rat liver microsomal preparations. *Biochem. J.*, **174**, 839.

Neal, G. E. & Colley, P. J. (1979) The formation 2,3-dihydroxy aflatoxin $B_1$ *in vitro* by rat liver microsomes. *FEBS Lett.*, **101**, 382.

Rutenberg, A. M., Kim, H., Fischbein, J. W., Hanker, J. S., Wasserkrug, H. L. & Seligman, A. M. (1969) Histochemical and ultrastructural demonstration of γ-glutamyl transpeptidase activity. *J. Histochem. Cytochem.*, **17**, 517.

Schaeffer, W. I. & Heintz, N. H. (1978) A diploid rat liver cell culture. IV. Malignant transformation by aflatoxin $B_1$. *In Vitro*, **14**, 418.

Shinozuka, H. & Lombardi, B. (1980) Synergistic effect of a choline-devoid diet and phenobarbital in promoting the emergence of foci of γ-glutamyl transpeptidase-positive hepatocytes in the liver of carcinogen-treated rats. *Cancer Res.*, **40**, 3846.

Sirica, A. E., Richards, W., Tsukada, Y., Sattler, C. A. & Pitot, H. C. (1979) Fetal phenotypic expression by adult rat hepatocytes on collagen gel/nylon meshes. *Proc. Natl Acad. Sci.*, **76**, 283.

Smith, D. G., Ding, J. L. & Peters, T. J. (1979) A sensitive fluorimetric assay for γ-glutamyl transferase. *Anal. Biochem.*, **100**, 136.

Solt, D. & Farber, E. (1976) New principle for the analysis of chemical carcinogenesis. *Nature*, **263**, 701.

Solt, D. B., Medline, A. & Farber, E. (1977) Rapid emergence of carcinogen-induced hyperplastic lesions in a new model for the sequential analysis of liver carcinogenesis. *Am. J. Pathol.*, **88**, 595.

Tate, S. S. & Meister, A. (1978) Serine–borate complex as a transition-state inhibitor of γ-glutamyl transpeptidase. *Proc. Natl Acad. Sci.*, **75**, 4806.

Toyoshima, K., Hiasa, Y., Ito, N. & Tsubura, Y. (1970). *In vitro* malignant transformation of cells derived from rat liver by means of aflatoxin $B_1$. *Gann*, **61**, 557.

Tsuchida, S., Hoshino, K., Sato, T., Ito, N. & Sato, K. (1979) Purification of γ-glutamyltransferases from rat hepatomas and hyperplastic hepatic nodules and comparison with the enzyme from rat kidney. *Cancer Res.*, **39**, 4200.

Tsuda, H., Lee, G. & Farber, E. (1980) Induction of resistant hepatocytes as a new principle for a possible short-term *in vivo* test for carcinogens. *Cancer Res.*, **40**, 1157.

Williams, G. M., Elliott, J. M. & Weisburger, J. H. (1973) Carcinoma after malignant conversion *in vitro* of epithelial-like cells from rat liver following exposure to chemical carcinogens. *Cancer Res.*, **33**, 606.

Williams, G. M., Ohmori, T., Katayama, S. & Rice, J. M. (1980) Alteration by phenobarbital of membrane-associated enzymes including gamma glutamyl transpeptidase in mouse liver neoplasms. *Carcinogenesis*, **1**, 813.